# Beyond the diagnostic code: what two million births can and cannot tell us about maternal sleep and offspring neurodevelopment

**DOI:** 10.1007/s44470-026-00142-3

**Published:** 2026-10-02

**Authors:** Po-Yang Tsou, Ignacio E. Tapia

**Affiliations:** 1 Division of Pulmonary Medicine, Boston Children’s Hospital, Harvard Medical School, Boston, MA USA; 2 Division of Sleep and Circadian Disorders, Brigham and Women’s Hospital, Harvard Medical School, Boston, MA USA; 3 Division of Pediatric Pulmonology, University of Miami Health System, Miami, FL USA; 4 Batchelor Children’s Research Institute, 1580 NW 10th Ave., 1st Floor, Miami, Fl 33136 USA

Sleep disturbance iscommon in pregnancy. Insomnia, restless legs syndrome, and sleep-disordered breathing each commonly emerge or worsen as pregnancy advances [1–3]. Whether maternal sleep disorders affect the developing fetal brain is biologically plausible: intermittent hypoxia, glucocorticoid signaling, and systemic inflammation each offer a mechanism by which disrupted maternal sleep could alter fetal neurodevelopment. However, whether this association translates to measurable neurodevelopmental outcomes in childhood is unknown. Prior studies have been constrained by small samples, short follow-up, and questionnaire-based exposure ascertainment, and restricted to perinatal or infancy endpoints. In their study recently published in the Journal of Clinical Sleep Medicine, Oh and Song address these gaps using a large and reasonably well-characterized national birth cohort [4].

Oh and Song analyzed 1,993,494 live births in South Korea’s National Health Insurance Service database between 2008 and 2012, identifying maternal sleep disorders from ICD-10 diagnostic codes and ascertaining long-term neurodevelopmental disorders (LNDD; ICD-10 F70–F95) through 2023, for up to 15 years of follow-up. Approximately 1.7% of mothers carried a coded sleep disorder diagnosis, reflecting clinically recognized disease rather than true prevalence, which is considerably higher. The study’s scale establishes the association’s robustness. After 1:5 propensity-score matching, maternal sleep disorder was associated with a higher risk of any LNDD (hazard ratio [HR] 1.36; 95% CI, 1.32–1.41), with a cumulative incidence of 13.3% versus 9.9%. Importantly, the association was consistent across intellectual, developmental, and behavioral diagnoses, and stronger when the disorder was diagnosed before than during pregnancy (HR 1.31 vs 1.18). It persisted in singleton and nulliparous births and across strata of maternal psychiatric and obstetric comorbidity. A signal this consistent is not easily explained by chance or a single confounder. Long suggested by smaller studies but never powered to confirm, the association can now be regarded as a robust population-level signal. Earlier cohorts have been small or limited to perinatal endpoints [5–7]; and nationwide analyses have so far addressed single conditions, such as maternal sleep apnea [8, 9]. The present work addresses these gaps simultaneously, leveraging population-scale data to examine a broad spectrum of maternal sleep disorders and neurodevelopmental outcomes across childhood. However, the meaning remains open to interpretation, as the design enabling this scale simultaneously limits its data inferences.

The first limitation is intrinsic to the administrative database design: maternal sleep disorder is defined by a diagnostic code, not a phenotype. Even the apnea–hypopnea index, if available, would only capture severity rather than a phenotype as obstructive sleep apnea ranges from a sleepy, symptomatic presentation to a minimally symptomatic one. Insomnia is equally diverse. The exposure is also heterogeneous: insomnia accounts for roughly two-thirds of the diagnosed group and likely skews the overall results. In contrast, sleep apnea (*n* = 388) and restless legs syndrome (*n* = 453) are far less common and mechanistically distinct. Moreover, rarer subtypes are too sparse to interpret (parasomnia, *n* = 17). A code also requires maternal recognition of symptoms, seeking, and receiving care. However, since disrupted sleep is so often normalized in pregnancy, many affected mothers never receive care. This misclassification can bias estimates in either direction. Undiagnosed but affected mothers in the comparison group dilute the exposure contrast and attenuate associations toward the null, while more thorough outcome ascertainment among coded mothers could introduce bias in the opposite direction. Importantly, the exposure that matters biologically is not the diagnosis itself but its physiological correlates: severity, intermittent hypoxia or fragmentation, and cumulative burden. Capturing physiologic measurements is the step that would move an observed association toward a mechanism, and ultimately toward a target for intervention.

The authors reasonably urge early identification and management. However, transitioning from a known association to an effective fetal-protective treatment is highly complex, presenting significant clinical and statistical hurdles. Even where treatment clearly helps, its benefits may align more closely with a patient’s specific physiology than their formal diagnosis. For example, major CPAP trials showed that cardiovascular benefits were heavily concentrated in participants with the highest baseline cardiovascular risk physiology [10]. Comorbidity further compounds this: in practice, starting CPAP while leaving coexisting restless legs syndrome untreated seldom succeeds, as the disorders interact and adherence fails. A diagnostic code cannot anticipate this issue. The constructive next step is to therefore use study designs that permit cleaner causal comparison, such as treated-versus-untreated cohorts phenotyped at baseline, sibling and cousin comparisons that hold family background fixed [11], and dose–response analyses keyed to severity and timing. Treatment options are also narrower in pregnancy: dopaminergic agents and opioids for restless legs are avoided and several hypnotics discouraged, while CPAP remains among the few clearly acceptable options for sleep apnea [12]. For now, the evidence that treating maternal sleep disorders benefits the fetus remains unfortunately inconclusive.

This study opens the door to future work. Recognition is the first priority. The heavily suppressed 1.7% coding rate, compared to a much higher true prevalence, reflects both administrative data constraints and a systemic tendency to overlook maternal sleep health. Prospective ascertainment is essential to finally identify this underserved, uncounted population. The second step is understanding the underlying mechanisms, as pregnancy drives these disorders through distinct pathways: anatomical airway and weight changes contribute to sleep apnea, iron depletion to restless legs syndrome, and hyperarousal to insomnia. Objective measurements would capture the physiology a code omits, for apnea the hypoxic burden that animal models already tie to autism-relevant phenotypes in offspring [13]. The third step is treatment, limited by low awareness and the narrow range of therapies available in pregnancy. Confirming a downstream fetal benefit is a far greater challenge, requiring precise disorder characterization, multi-year developmental tracking, and the isolation of treatment effects from confounding variables. By successfully establishing the first signal, this study takes the essential first step.

## Data Availability

Not applicable.
